# Probiotic *Bacillus coagulans* GBI-30, 6086 reduces exercise-induced muscle damage and increases recovery

**DOI:** 10.7717/peerj.2276

**Published:** 2016-07-21

**Authors:** Ralf Jäger, Kevin A. Shields, Ryan P. Lowery, Eduardo O. De Souza, Jeremy M. Partl, Chase Hollmer, Martin Purpura, Jacob M. Wilson

**Affiliations:** 1 Increnovo LLC, Milwaukee, WI, United States of America; 2 Department of Health Sciences and Human Performance, University of Tampa, Tampa, FL, United States of America; 3 Research Division, Applied Science and Performance Institute, Tampa, FL, United States of America

**Keywords:** Gut-muscle-axis, Probiotics, Protein utilization, Athletic performance, Sports nutrition

## Abstract

**Objective.** Probiotics have been reported to support healthy digestive and immune function, aid in protein absorption, and decrease inflammation. Further, a trend to increase vertical jump power has been observed following co-administration of protein and probiotics in resistance-trained subjects. However, to date the potential beneficial effect of probiotics on recovery from high intensity resistance exercise have yet to be explored. Therefore, this study examined the effect of co-administration of protein and probiotics on muscle damage, recovery and performance following a damaging exercise bout.

**Design.** Twenty nine (*n* = 29) recreationally-trained males (mean ± SD; 21.5 ± 2.8 years; 89.7 ± 28.2 kg; 177.4 ± 8.0 cm) were assigned to consume either 20 g of casein (PRO) or 20 g of casein plus probiotic (1 billion CFU * Bacillus coagulans* GBI-30, 6086, PROBC) in a crossover, diet-controlled design. After two weeks of supplementation, perceptional measures, athletic performance, and muscle damage were analyzed following a damaging exercise bout.

**Results.** The damaging exercise bout significantly increased muscle soreness, and reduced perceived recovery; however, PROBC significantly increased recovery at 24 and 72 h, and decreased soreness at 72 h post exercise in comparison to PRO. Perceptual measures were confirmed by increases in CK (PRO: +266.8%, *p* = 0.0002; PROBC: +137.7%, *p* = 0.01), with PROBC showing a trend towards reduced muscle damage (*p* = 0.08). The muscle-damaging exercise resulted in significantly increased muscle swelling and Blood Urea Nitrogen levels in both conditions with no difference between groups. The strenuous exercise significantly reduced athletic performance in PRO (Wingate Peak Power; PRO: (−39.8 watts, −5.3%, *p* = 0.03)), whereas PROBC maintained performance (+10.1 watts, +1.7%).

**Conclusions.** The results provide evidence that probiotic supplementation in combination with protein tended to reduce indices of muscle damage, improves recovery, and maintains physical performance subsequent to damaging exercise.

## Introduction

Microbiota composition and diversity positively correlates with protein intake and exercise and is linked to a more favorable inflammatory and metabolic profile (Clarke et al., 2014). While moderate intensity exercise reduces infection risk, high intensity exercise actually increases infection risk. Immune suppression in athletes worsens by psychological stress, environmental extremes, exposure to large crowds, or increased exposure to pathogens due to elevated breathing during exercise (Nieman, 1997). Approximately 7.2–8.9% of athletes of recent Olympic Summer and Winter games reported an illness, of which 46–58% of those illnesses being infections (Engebretsen et al., 2015; Engebretsen et al., 2013). Probiotics have been reported to reduce the number, severity, and duration of upper respiratory infections and gastrointestinal distress in athletes (Gleeson et al., 2011; Salarkia et al., 2013; Haywood et al., 2014; Cox et al., 2010). However, some intervention studies in athletes have shown no beneficial effect of probiotics on immune and gastrointestinal health (Tiollier et al., 2007; Gleeson et al., 2012), raising questions of strain-specific benefits, optimal and minimal duration of supplementation and dose, training intensity and even gender specificity (West et al., 2011). While one study has shown potential performance benefits of probiotics under extreme environmental conditions (Shing et al., 2014), there is currently no evidence that concludes that probiotics may directly enhance athletic performance (Cox et al., 2010; West et al., 2011; Lamprecht et al., 2012).

In a recent pilot study from our laboratory, a trend towards an increase in vertical jump power was noted. Vertical jump power was greater following 8 weeks of full body workouts 4-times per week daily while ingesting *Bacillus coagulans* GBI-30, 6086 and protein daily compared to protein alone (Georges et al., 2014). The spore-forming probiotic *Bacillus coagulans* GBI-30, 6086 (Keller et al., 2010) has been reported to support healthy digestive (Kalman et al., 2009) and immune function (Kimmel et al., 2010), including increased protein absorption (Matthuis, Keller & Farmer, 2010). Protein supplementation improves recovery and training adaptations, resulting in an increase in lean body mass with subsequent greater gains in strength and power (Campbell et al., 2007). We speculate that the beneficial effects observed in vertical jump performance might be based on aiding muscle recovery through gut microbial modulation. Thus, the purpose of this investigation was to determine if the co-administration of *Bacillus coagulans* GBI-30, 6086 with protein has a beneficial effect on muscle damage, performance, and recovery following a damaging exercise bout.

## Materials and Methods

### Subjects characteristics

Forty six male subjects with at least 3 months of recreational training from the University of Tampa and surrounding areas (Tampa, FL, USA) were screened for eligibility of which 12 were excluded (three did not meet inclusion criteria, five declined to participate, four for other reasons) from participation. Thirty four subjects were allocated to intervention of which one subject did not report for pre testing, two subjects did not report for post testing and two subjects were lost due to time commitment. Twenty nine (*n* = 29) recreationally trained males (mean ± SD; 21.5 ± 2.8 years; 89.7 ± 28.2 kg; 177.4 ± 8.0 cm) completed this study. Each participant gave written informed consent before participating in the study. This study was approved by the University of Tampa Institutional Review Board and registered with ISRCTN (ISRCTN21076380). Exclusion criteria included the use of prescription medications (including anti-inflammatory agents, antibiotic treatment), ergogenic supplements, probiotics or digestive enzymes within the previous two months, or suffering from any skeletomuscular, medical, or metabolic contraindications.

### Experimental design

**Figure 1:**
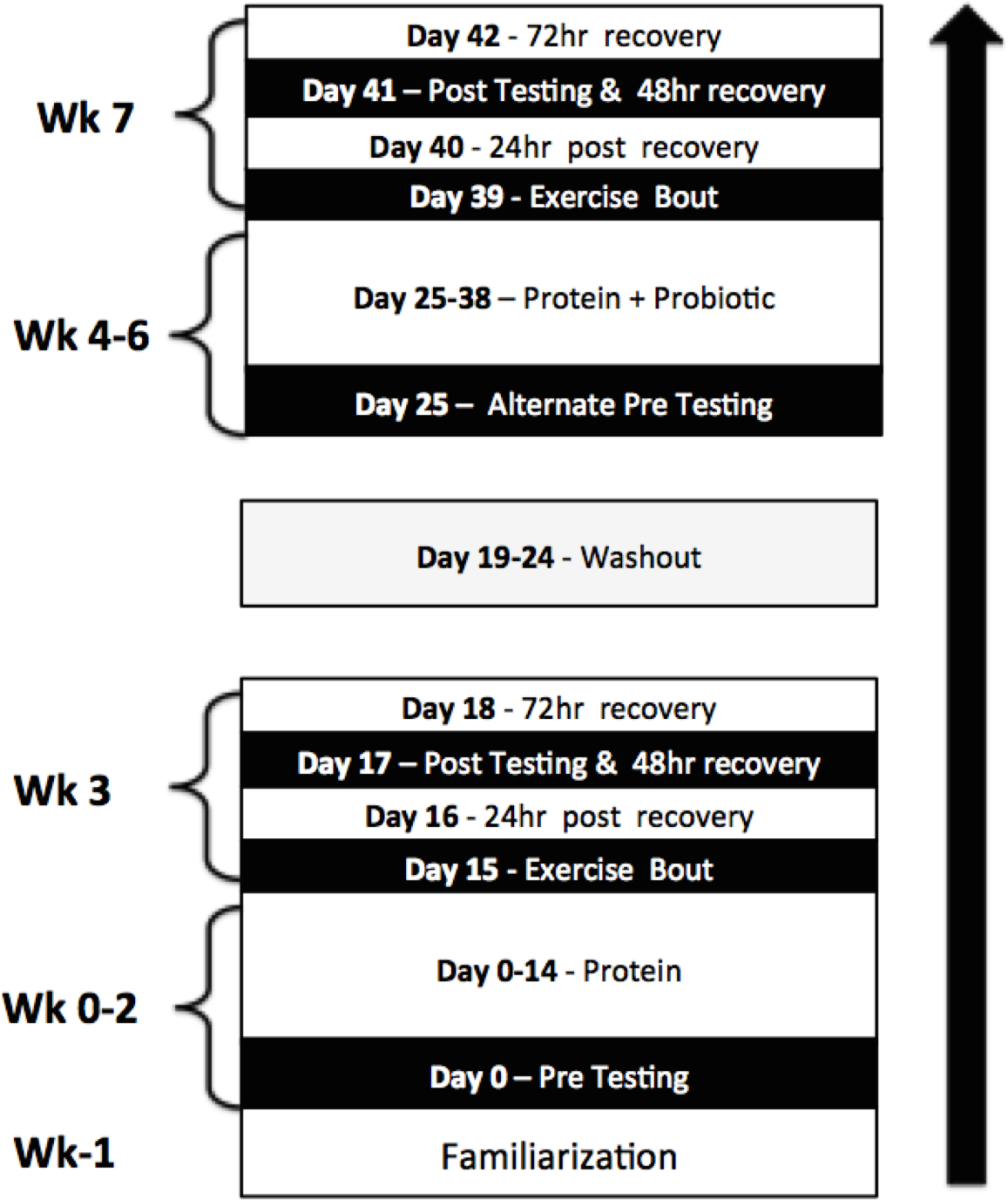
Study design.

To examine the effects of co-administration of *Bacillus coagulans* GBI-30, 6086 with protein a 7-week, placebo and diet-controlled, repeated measures study design was employed. Given the lack of data concerning the wash-out period necessary for gut microbiota following probiotic supplementation a crossover design was not employed. Participants took part in two performance test familiarization sessions prior to beginning supplementation. Thereafter, baseline testing was performed which included measures of muscle soreness and recovery, blood sampling for markers of inflammation, and performance testing. Participants then supplemented with protein (PRO) for 14 days. At the conclusion of the supplementation periods, participants performed the experimental protocol which included a muscle damaging one-legged exercise bout followed by measurements of outcomes of interest. Perceived recovery and muscle soreness was measured 1, 2, and 3 days post exercise. In addition, participant’s strength and power, as well as creatine kinase (CK) and blood urea nitrogen (BUN) levels, and muscle thickness were measured 2 days post exercise. A week wash-out period then ensued followed by 14 days of supplementation with the co-administration of *Bacillus coagulans* GBI-30, 6086 with protein (PROBC). Participants used the contralateral leg during the second muscle-damaging exercise to prevent the repeated bout effect (Connolly, Reed & McHugh, 2002). The sequence in which the dominate leg went first or second was randomized. Figure 1 depicts a timeline of the major events of the study.

#### Exercise bout

After 1RMs were determined, participants took part in a single-leg exercise training protocol. The exercise bout consisted of three exercises; all sets and repetitions of the first exercise were performed completely before proceeding to the next exercises. Exercise 1 was performed independently while Exercises 2 and 3 were performed back-to-back (superset) (See Table 1). Between each set and exercise, participants were allotted 60 and 120-second rest periods, respectively. If participants failed to complete a set at the prescribed weight, weight was decreased in 5–10% increments until all prescribed reps could be completed without assistance. All exercise sessions were monitored by a Certified Strength and Conditioning Specialist and supervised by trained laboratory technicians.

**Table 1:** Single-leg exercise bout protocol.

Exercise name	Sets × reps	Rest time	% of 1RM
1. Single-leg leg press	10 × 10	60 s	70
2. Leg extension	5 × 12	60 s	∼65
3. Rear foot elevated split squat	5 × 12	60 s	X

#### Supplementation and dietary monitoring

In a single-blind fashion participants were assigned to consume 20 g casein, and following a one week wash-out period 20 g of casein plus 1 billion CFU *Bacillus coagulans* GBI-30, 6086 (Ganeden**BC**^30^; Ganeden Biotech Inc., Mayfield Heights, OH, USA) daily for 2 weeks. Participants received no supplementation during the washout period. Both supplemental conditions were identical in appearance, taste, and weight. Participants were instructed to consume each serving accompanying breakfast. At pre and post-testing visits (Day 17 and 26), participants came into the laboratory fasted and were then provided with a standardized meal consisting of 190 kcal (FAT: 6 g, CHO: 29 g, PRO: 4 g), 30 min prior to their workout.

Supplementation compliance was measured by the collection of supplement containers at the end of each 7-day testing period. For dietary analysis, participants were required to log their dietary intake for three days prior to the first testing trial. The three-day dietary recalls were tracked in MyFitnessPal™ where macronutrient consumption was averaged and assigned to participants for their dietary intake for the duration of the study. Participants experienced no adverse events while taking the supplements. Subject compliance was 98% across conditions.

### Experimental protocol

#### Blood sampling

Fasted (at least 8 h) blood samples were collected from participants in the seated upright position. Fingers were cleaned with a standard alcohol prep pad (AeroMed, Glastonbury, CT, USA) and blood was collected from a 1.75 mm incision via Tenderlett™ (ITC, Edison, NJ, USA). Blood was collected into a 125 µL Safe-T-Fill Mini Capillary Blood Collection System (RAM Scientific Inc., Germany) which was then transferred by a pipette (Eppendorf, Hauppauge, NY) with 200 µL pipette tips (Neptune Scientific, San Diego, CA, USA) into Metalyte 8 Panel (Abaxis Inc., Union City, CA, USA). The Metalyte panels were run through a Piccolo Xpress Machine (Abaxis Inc., Union City, CA, USA) by a trained technician for determination of CK and BUN. The intra assay variance was less than 3% for all analytes.

#### Muscle thickness

Two-dimensional, B-mode ultrasonography (General Electric Company LOGIQ *e*, Milwaukee, WI, USA) was used to determine muscle thickness of the vastus lateralis (VL). Muscle thickness was measured at 50% femur length (the distance from the greater trochanter to the lateral epicondyle of the knee) along the lateral portion of the VL on the right leg. The spot was marked with a permanent marker and participants were instructed to keep their mark throughout the duration of the study in order to maintain consistency of the measurement site. An 8 MHz scanning transducer was oriented perpendicular to muscle belly of the VL. Water-soluble transmission gel was applied to the scanning transducer to aid acoustic coupling and prevent direct contact with the skin that could pressurize and deform the underlying tissues. To obtain images, participants lay supine with fully extended legs and muscles relaxed. When a visible image was projected on the monitor, the image was frozen. Muscle thickness measurements were extrapolated from the monitor screen by measuring the distance from the interface of the muscle tissue and subcutaneous fat to the surface of the femur. The same researcher performed all ultrasound assessments and was blinded to the treatment groups. Muscle thickness of the VL was assessed at baseline and the completion of the study. The CV for VL muscle thickness measurements was 2%. The change in muscle thickness measured using ultrasound is the index for muscle swelling due to damage.

#### Power assessment

Anaerobic power was assessed via Monark Wingate cycle ergometry (Monark™, Vansbro, Sweden) using a modified Wingate test. During the cycling test, the participant was instructed to cycle against a predetermined resistance (7.5% of body mass) as fast as possible for 10 s. The saddle height was adjusted for each participant in order to produce a 5–10° knee flexion while the foot was in the low position of the central void. A standardized verbal stimulus was provided to the participant. Power output was recorded in real time by a computer connected to the Monark standard cycle ergometer (Monark model 894e, Vansbro, Sweden) during a 10-second sprint test. Wingate PP was recorded using Monark Anaerobic test software (Monark Anaerobic Wingate Software, Version 1.0, Monark, Vansbro, Sweden). Power measurements were recorded in watts and were conducted at baseline and the completion of the study. The CV for PP were 4.0%. Single-leg vertical jump was measured via Tendo Unit (Trencin, Slovak Republic). The participant was instructed to perform a single-leg countermovement jump as fast and as high as they could. The highest of the three attempts was recorded for Peak Power (PP). Participants received 60 s between vertical jump attempts. In an attempt to eliminate the impact of instruction on technique, the only cue subjects were given was to perform a countermovement jump as high as possible.

#### Strength assessment

Strength was assessed via one repetition maximum testing (1RM) in the one-legged leg press. A research assistant that was certified by the National Strength and Conditioning Association (NSCA-CSCS) observed strength testing and loads were increased incrementally until maximal load or failure at a given load was reached. Briefly, participants performed a general warm-up and a specific warm-up consisting of two sets. During the first set, participants performed 10 repetitions with 50% of their predicted 1RM. For the second set, they performed five repetitions with 75% of the predicted 1RM. After the second warm-up, participants were instructed to rest for 3 min before attempting their 1RM. Following, each subject had 3 maximal attempts to achieve their 1RM load with 3–5 min rest between each attempt. Strong verbal encouragement was given throughout 1RM testing by the same investigator. The leg press was performed at a 45° in which attempts had to reach at least a 90° knee flexion angle to be deemed successful. The CV for 1RM testing which was acquired on two separate days separated by 72 h prior to the study was 3.4%. Data was recorded as the weight lifted, in kilograms. Strength measurements were conducted at pre and post testing.

#### Perceptual measures

The perceptual measures consisted of two visual analogue scales—rating of perceived recovery (PRS) and muscle soreness (VAS). Ratings PRS and VAS were recorded at pre testing (Week 0) and 24, 48, and 72 h after each exercise bout (Weeks 3 and 7). Both scales consisted of a value from 0 to 10. The PRS numbers between 0 and 2 consisted of being “very poorly recovered” anticipating declines in performance, between 4 and 6 being “adequately recovered” expecting similar performance, and between 8 and 10 being “well recovered” expecting increases in performance. The VAS numbers between 0 and 2 represented “no soreness”, between 4 and 6 represented “moderate soreness”, and between 8 and 10 represented “severe soreness.” Participants were asked to identify their level of perceived recovery and perceived soreness upon arrival to the laboratory (Sikorski et al., 2013).

### Statistical methods

Before carrying out the parametric statistical analysis, dependent variables were examined for a normal distribution and outliers through the investigation of boxplots and a normality test (i.e., Shapiro Wilks). If some dependent variables were considered not to be normally distributed, data was log-transformed and variables were investigated again. For creatine kinase (CK), some outliers were automatically removed by the software and the data was log-transformed. CK values that were more than 3 standard deviation units outside of the mean were considered as outliers and the researcher that performed analysis was blinded for the treatments. After that, CK was considered to be normally distributed and thus these log-transformed data were used in the subsequent statistical analysis. A two-way ANOVA with repeated measures was performed for dependent variables assuming time (baseline and post) and condition (PRO and PROBC) as fixed factors. Whenever a significant F-value was obtained, a post-hoc with Tukey’s adjustment was performed for multiple comparisons. In addition, we presented the mean value and confidence intervals of the absolute difference (CI_diff_). The confidence interval includes the value range in which the true population mean of the difference is likely to be. Positive and negative confidence intervals that did not cross zero were considered significant. The significance level was set at *p* ≤ 0.05, *p* values >0.05 and ≤0.10 were considered trends. Results are expressed as mean ± standard error unless otherwise stated.

**Figure 2:**
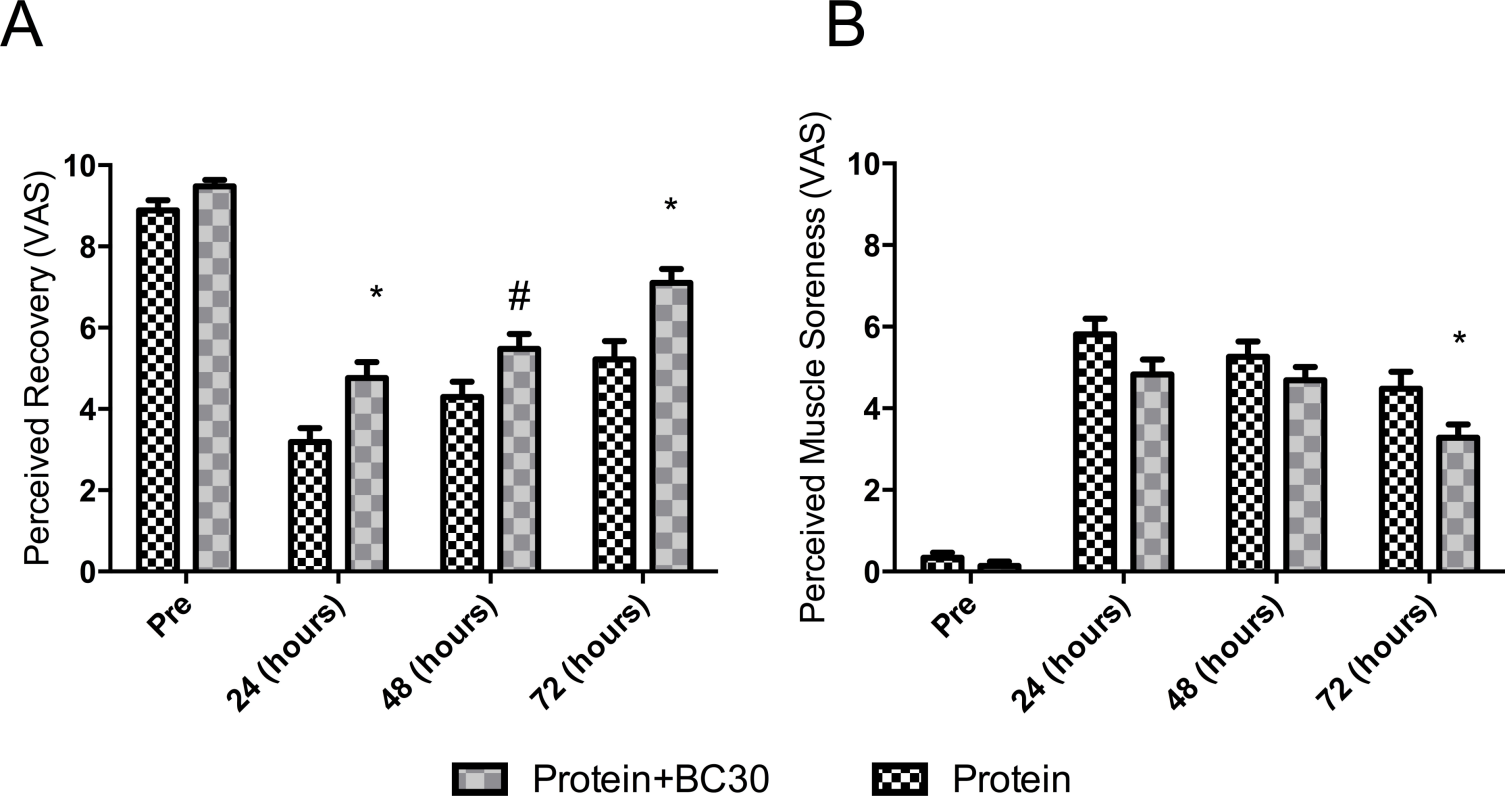
Changes in perceived recovery and muscle soreness (*indicates significantly different (*p* < 0.05), and ^#^indicates a trend (*p* = 0.06) between groups).

## Results

### Probiotics increase perceived recovery and reduce muscle soreness

Results for the perceived recovery status (PRS) and muscle soreness (VAS) are presented in Fig. 2. No significant between-conditions differences were detected prior to the experimental period (*p* > 0.05). Both conditions significantly reduced PRS across the time points (*p* < 0.0001). However, the between conditions analysis by CI_diff_ revealed that PROBC condition had higher recovery scores at 24 hours-post (e.g., PROBC vs PRO: mean 1.55 AU, 95% CI_diff:_ [0.34–2.79AU]) and at 72 hours-post (e.g., PROBC vs PRO: mean 1.88 AU, 95% CI_diff:_ [0.65–3.10AU]).

A similar response was observed for VAS. Both conditions significantly increased soreness perception across time (*p* < 0.02). However, analysis of CI_diff_ revealed that the PROBC condition had a lower average soreness score at 72 hours-post (e.g., PROBC vs PRO: mean −1.20 AU, 95% CI_diff:_ [−2.35–0.05 AU]).

### Probiotics decreased exercise-induced muscle damage

**Figure 3:**
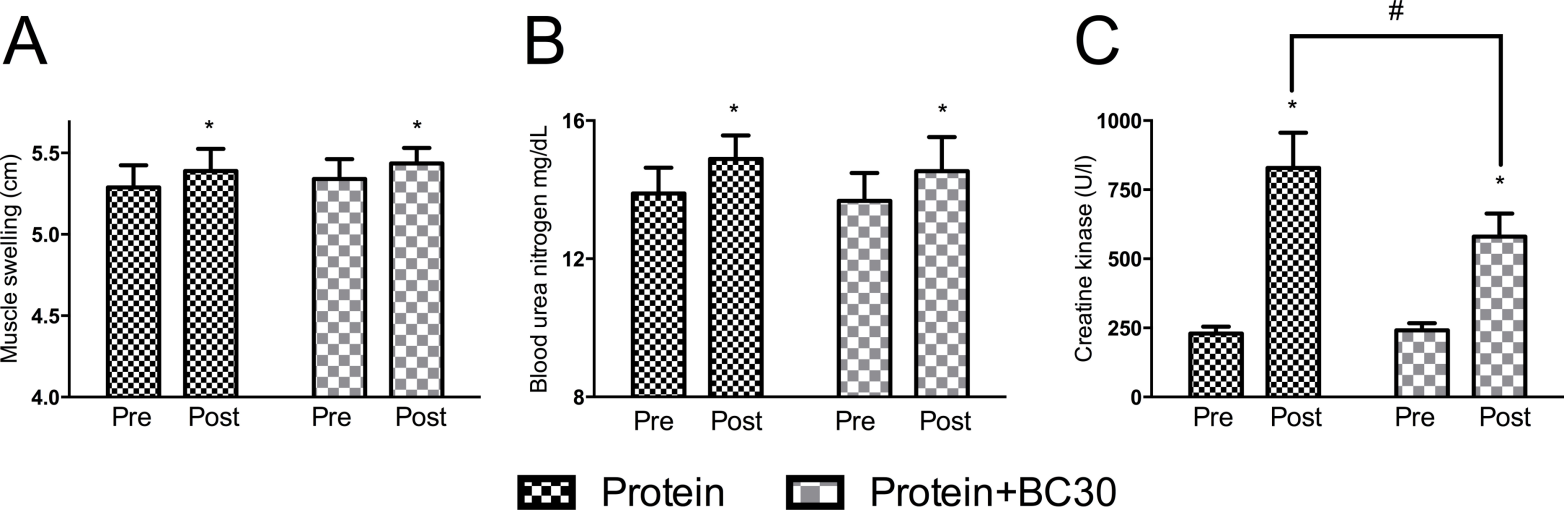
Changes in muscle swelling (A), BUN (B) and CK (C) (*indicates significantly (*p* < 0.05) different from pre-test, ^#^indicates trend (*p* = 0.08) between groups).

Results for blood parameters are presented in Fig. 3. Sample size for CK analysis after excluding outliers was *n* = 20. No significant between-condition differences in muscle swelling, blood urea nitrogen test (BUN) and creatine kinase (CK) were detected prior to experimental period (*p* > 0.05). Both conditions significantly increased muscle swelling and BUN (*p* < 0.005). However, for creatine kinase (CK), a trend toward a significant condition × time interaction was observed (*p* < 0.08). The PRO group demonstrated a relative increase of 261.2%, while the PROBC demonstrated a relative increase of 137.7% in serum CK levels.

### Probiotics prevents exercise-induced reduction in athletic performance

**Figure 4:**
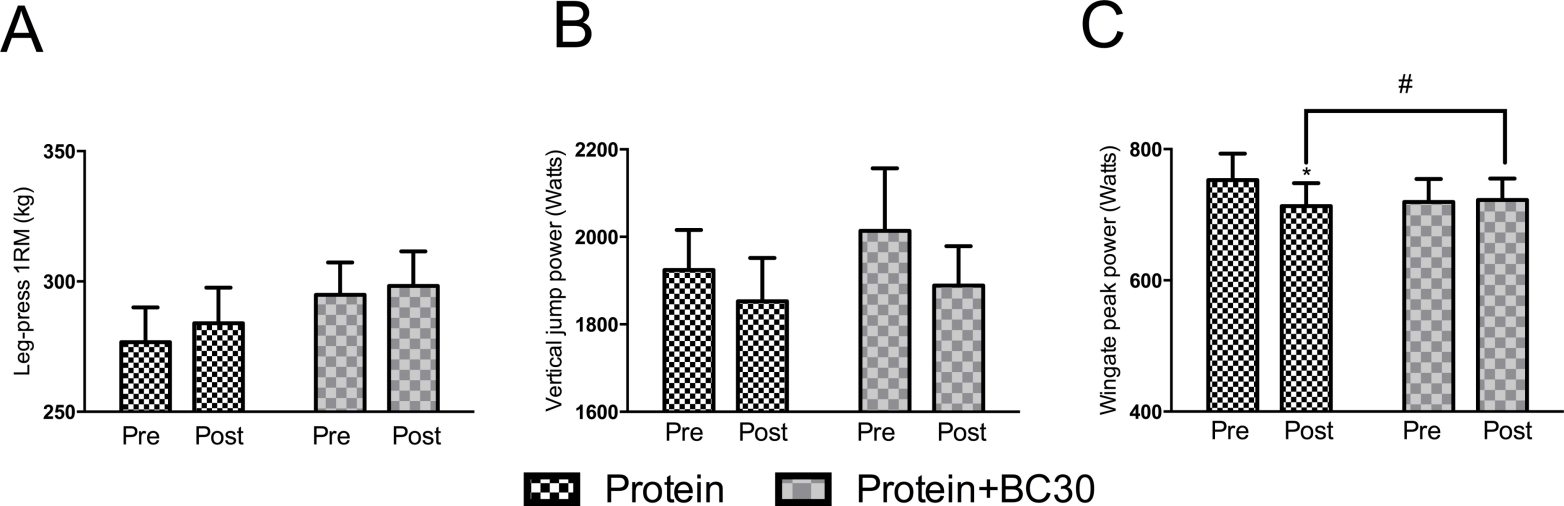
Changes in performance measures from pre to post testing (*indicates significantly (*p* < 0.05) different from pre-test, ^#^indicates trend (*p* = 0.07) between groups).

Results for performance variables are presented in Fig. 4. No significant between-conditions differences in 1RM leg-press, vertical jump power and Wingate power were detected prior to the experimental period (*p* > 0.05). For 1RM leg-press and vertical jump power, there were no significant changes within and between conditions (*p* > 0.05).

For Wingate power, a trend towards a significant condition × time interaction was observed (*p* < 0.07). However, the CI_diff_ revealed a between-condition difference which demonstrated a significant decrease (−5.3%) in Wingate power: PRO: mean −39.7 Watts, 95% CI_diff:_ [−77.98–−2.6]. The PROBC condition had no significant change in Wingate power (+1.7%): PROBC: mean 3.08 Watts, 95% CI_diff:_ [−35.08–41.25] Watts.

## Discussion

Exhaustive or intense resistance training is often accompanied by acute increases in muscle damage as well as delayed onset muscle soreness (DOMS), which results in decreased performance. However, in order to increase athletic performance, the training must be challenging enough to make adaptations to resistance training stimuli. The mechanism of exercise-induced muscle damage includes mechanical and metabolic pathways and the magnitude of damage depends on the type, the intensity and duration of the exercise, as well as the nutritional status (Sousa, Teixeira & Soares, 2014).

Pre- and post-exercise protein consumption increases muscle hypertrophy, exercise recovery and enhances immune functions during periods of intense training (Campbell et al., 2007). Athletes can choose from numerous different protein sources, which differ in amino acid composition, bioavailability and absorption kinetics (Joy et al., 2013). Stimulation of muscle protein synthesis is greater after the consumption of fast digesting proteins (whey) in comparison to slow digested proteins (casein) (Tang et al., 2009), and whey protein results in greater gains in strength and lean body mass, when compared to casein (Cribb et al., 2006). Improving the digestibility of protein results in faster recovery of strength after muscle damaging exercise (Buckley et al., 2010), and increases glycogen levels after exercise, which facilitates recovery and repair (Kanda et al., 2012).

*Bacillus coagulans* produce digestive enzymes (Wang & Gu, 2010) that are active under gut conditions (alkaline proteases, etc.) and these proteases have been shown to digest proteins more efficiently than the endogenous human proteases alone (Minevich et al., 2015). *Bacillus coagulans* GBI-30, 6086 enhances the health of the cells of the gut lining improving nutrient absorption including minerals, peptides and amino acids by decreasing inflammation and encouraging optimum development of the absorptive area of the villi (Kimmel et al., 2010).

The main findings of the current study were that the muscle damaging exercise resulted in significantly reduced perceived recovery and increased muscle soreness, increased muscle damage and reduced performance (Wingate peak power) in the protein supplemented group. The co-administration of *Bacillus coagulans* GBI-30, 6086 and protein significantly increasing recovery 24 and 72 h, and muscle soreness 72 h after exercise. The addition of the probiotic to the protein tended to reduced muscle damage and prevented the decline in peak power. Strength or vertical jump power did not significantly decline in both groups, indicating that the exercise protocol was not challenging enough to negatively influence those measures.

### Future probiotic research in athletes

Probiotics have been linked to numerous health benefits potentially relevant to athletes including normalizing age related drops in testosterone levels (Poutahidis et al., 2014), increasing in neurotransmitter synthesis (Lyte, 2011), reducing stress-induced cortisol levels (Bravo et al., 2011), inflammation (Klemenak et al., 2015) or improving mood (Steenbergen et al., 2015). However, all these potential benefits lack current substantiation in human intervention trials in an athletic population.

### Limitations

*Bacillus coagulans* GBI-30, 6086 supplementation has been shown to increase populations of beneficial bacteria (Nyangale et al., 2014). However, the wash-out period to reset the microbiota to baseline levels is currently unknown. In order to prevent a potential carry-over effect from the probiotic supplementation, a single-blind design was chosen, starting supplementation with the protein group. If improved recovery and reduced muscle damage to an acute exercise bout and the subsequent increase in performance will result increases in lean body mass, strength and power needs to be confirmed in a long-term training study.

### Conclusion

In conclusion, the probiotic supplement tended to protect the muscle from damage and may have helped recovery of physical performance. Further studies should investigate the relationship between direct and indirect (through improved protein utilization) effects of probiotic supplementation and will provide further insights into strategies to influence the gut microbiota to optimize muscle health, specifically in an aging population with impaired nutrient utilization (sarcopenia).

##  Supplemental Information

10.7717/peerj.2276/supp-1 Data S1 Raw data

## Additional Information and Declarations

### Data Availability

The following information was supplied regarding data availability: The raw data has been supplied as Data S1.

### Clinical Trial Registration

The following information was supplied regarding Clinical Trial registration: ISRCTN21076380.
